# Atypical ‘long-tailed’ cockroaches arose during Cretaceous in response to angiosperm terrestrial revolution

**DOI:** 10.7717/peerj.15067

**Published:** 2023-03-29

**Authors:** Xin-Ran Li, Di-Ying Huang

**Affiliations:** State Key Laboratory of Palaeobiology and Stratigraphy, Center for Excellence in Life and Paleoenvironment, Nanjing Institute of Geology and Palaeontology, Chinese Academy of Sciences, Nanjing, China

**Keywords:** Amber, Angiosperm, Dictyoptera, Eoblattodea, Fossil, Holopandictyoptera, Ovipositor, Roachoids

## Abstract

Typical cockroaches are flat, broad, with large pronotum and wings covering the body. This conserved morphotype dates back to the Carboniferous, during which the ancestral cockroaches, or roachoids, originated. On the other hand, the ovipositor of cockroaches gradually reduced during the Mesozoic, coupled with a major shift of reproductive strategy. By the Cretaceous, long external ovipositors became rare, most cockroaches used very short or even hidden internal ovipositors to fabricate egg cases (oothecae), which is an innovation for egg protection. Here, we describe two cockroaches from mid-Cretaceous Myanmar amber: *Ensiferoblatta oecanthoides* gen. et sp. nov. (Ensiferoblattidae fam. nov.) and *Proceroblatta colossea* gen. et sp. nov. They are slim, elongate, fusiform, with longitudinal pronotum, and have long external ovipositors. The combination of these traits represents a unique morphotype, which resembles crickets and katydids (Ensifera) more than general cockroaches. *Ensiferoblatta* and *Proceroblatta* may be arboreal, feeding on and/or laying eggs into certain angiosperms that newly emerged. Their open habit causes latent impairment to viability, and may contribute to their extinction. These new taxa are the youngest members of the ancient, extinct group of cockroaches, namely Eoblattodea, which are characterized by long ovipositors. We speculate that the extinction of certain gymnosperm hosts almost ended the 200-My triumph of Eoblattodea. Despite an attempt to adapt to angiosperm hosts, *Ensiferoblatta*, *Proceroblatta* and suchlike cockroaches as an evolutionary dead end failed to save Eoblattodea from extinction. The lack of protection for eggs (maternal care in particular) might accelerate the extinction of Eoblattodea as a whole.

## Introduction

Cockroaches are common insects that inhabit all around tropical and temperate regions, with ca. 4,000 extant species (Beccaloni, 2014). Household species such as American cockroaches (*Periplaneta americana* (L.)) and German cockroaches (*Blattella germanica* (L.)) give people the typical impression of cockroaches: flat, broad, with large shield-like pronotum and oval tegmina covering body and agile legs. This typical shape of cockroaches is adaptive to living and sheltering in crevices and loose substrates (Bell, Nalepa & Roth, 2007). Most of cockroach fossils with body preserved are in the typical shape. Exceptions include beetle-like ones and elongate ones. Beetle-like fossil cockroaches are represented by Umenocoleidae and Cratovitismidae (Bechly, 2007; Lee, 2016; Beutel, Luo & Wipfler, 2020; Luo, Xu & Jarzembowski, 2020; Luo et al., 2021; Luo et al., 2022), which are comparable with extant *Diploptera* cockroaches in exposed head, smaller pronotum, and heavily sclerotized forewings (especially *D. maculata* and *D. elliptica*, see Li & Wang, 2015; Li, Li & Wang, 2017). Fossil cockroaches with elongate and agile appearance are represented by Raphidiomimidae and Manipulatoridae (Vishnyakova, 1973; Liang, Vršanský & Ren, 2012; Vršanský & Bechly, 2015; Li & Huang, 2022), which have elongate head and appendages in addition to the long body build. In spite of some debates, little is known about the habits and habitats of these atypical fossil cockroaches.

The oviposition behaviour of fossil cockroaches is also little known. The properties of the ovipositor directly correlate with reproductive strategy, the shift of which holds a key position in the evolutionary history of cockroaches. It is believed that the external ovipositor of ancient cockroaches had gradually shortened and eventually became hidden inside the abdomen (Laurentiaux, 1959; Vishnyakova, 1980; Roth, 2003; Anisyutkin & Gorochov, 2005; Grimaldi & Engel, 2005; Hörnig et al., 2018; Li, 2019). As with the ovipositor shortening and being concealed, cockroaches developed an ootheca to protect the eggs (Roth, 1968), and this is the very reproductive strategy of extant cockroaches. As far as we know, no cockroach fossil with an external ovipositor was found from the Cenozoic, whereas many were found from the Jurassic and Cretaceous. During the Jurassic, ovipositors in various length co-occurred: some are very long, and likely blade-like or sword-shaped (*e.g.*, Vishnyakova, 1968; Liang, Shih & Ren, 2017), some extend only a short distance outside the abdomen (*e.g.*, Vishnyakova, 1968; Liang, Vršanský & Ren, 2012), and others are intermediate. In comparison, Cretaceous cockroaches mostly have internal or shortly exposed ovipositors (*e.g.*, Anisyutkin & Gorochov, 2008; Lee, 2016; Qiu, Wang & Che, 2019; see also the closely related Alienoptera in Bai et al., 2018), with a few exceptions (Vishnyakova, 1986; Ren et al., 1995, but therein dated to Jurassic). This implies that the evolution of the ovipositor was towards reduction.

Here we describe new cockroach fossils from the mid-Cretaceous Myanmar amber. These cockroaches represent a unique, tree-cricket-like morphotype, which implies arboreal lifestyle associated with the angiosperm terrestrial revolution. Their long, sabre-shaped ovipositors, which were known from impression fossils only, demonstrates that long-ovipositored cockroaches survived to ca. 100 Mya at least, leaving a 200 My history of success without fabricating an egg case. In addition, the extinction of long-ovipositored cockroaches including this morphotype is briefly discussed.

## Materials & Methods

### Source and depository of materials

The cockroaches are preserved in amber pieces collected from deposits in the Hukawng Valley of northern Myanmar. Shi et al. (2012) dated Myanmar amber at 98.79 ± 0.62 Mya based on zircon U-Pb SIMS. Owing to methodological limits, the zircon U-Pb SIMS age may be younger than the actual age by possibly more than 1% (Mao et al., 2018). Specimens are deposited at Nanjing Institute of Geology and Palaeontology (NIGP), Chinese Academy of Sciences, under accession numbers NIGP200821–200824.

### Specimen processing and imaging

To get a clearer view, the ambers were sanded with abrasive papers and polished with polishing powder. Photos were taken with a Zeiss AxioZoom V16 stereoscope; stacked using CombineZP 7.0 (by Alan Hadley, UK) and Photoshop CC 2015; and optimized using Photoshop CC 2015.

### Morphological description

Terminology largely follows Roth (2003) and Li et al. (2018). The unit of measurements is millimetre.

### Taxonomy

The electronic version of this article in Portable Document Format (PDF) will represent a published work according to the International Commission on Zoological Nomenclature (ICZN), and hence the new names contained in the electronic version are effectively published under that Code from the electronic edition alone. This published work and the nomenclatural acts it contains have been registered in ZooBank, the online registration system for the ICZN. The ZooBank LSIDs (Life Science Identifiers) can be resolved and the associated information viewed through any standard web browser by appending the LSID to the prefix http://zoobank.org/. The LSID for this publication is: urn:lsid:zoobank.org:pub:7015F012-0008-4885-BB51-C1340A0A3CA2. The online version of this work is archived and available from the following digital repositories: PeerJ, PubMed Central SCIE and CLOCKSS. The delimitation or definition of higher taxa is character-based, instead of conforming to the crown-stem group system, unless otherwise indicated. Paraphyletic taxa are valid, and sometimes superior to monophyletic ones by more efficient information retrieval and better referring to evolutionary grades, which are particularly important in the taxonomic and evolutionary studies on cockroaches (Li, 2019).

## Results

### Systematic palaeontology

**Table:** 

Class Insecta
Clade Holopandictyoptera Kluge, 2010 *sensu*Li, 2019
Plesiomorphon Eoblattodea Laurentiaux, 1959*sensu*Li, 2019
Family Ensiferoblattidae fam. nov. (LSID: urn:lsid:zoobank.org:act:A0CC40E4-8990-4CA5-9854-D1670E93EB11)
Type genus: *Ensiferoblatta* gen. nov.
Diagnosis. See the diagnosis of the type genus.

Apomorphies. Based on comparisons with typical Holopandictyoptera and Eoblattodea, in which most traits are deemed to be plesiomorphies, Ensiferoblattidae bear the following apomorphies: fusiform and slim body, exposed and comparatively large head, elongate pronotum, and elongate wings. Please note that these apomorphies are only provisional, and should be revised upon new observations from new materials.

### Genus *Ensiferoblatta* gen. nov.

(LSID: urn:lsid:zoobank.org:act:EA84E47F-47AD-4F79-AFF6-929484FCDDF3)

**Type species.** *Ensiferoblatta oecanthoides* gen. et sp. nov.

**Etymology.** Derived from Latin *ensifera*, sword-bearing, and *blatta*, cockroach. Feminine.

**Diagnosis (female only).** Body fusiform, not flat, length-width ratio significantly greater than the general build of cockroaches. Head nearly triangular, resembling the head of a mantis, hardly covered by pronotum, almost as wide as the full width of pronotum. Pronotum longer than width, widest at posterior fourth. Wings elongate as with body. Hindwing with fanwise folds. Each femur with a genicular spine, which is especially long in mid- and hindfemora. Both the anteroventral margin and the posteroventral margin with one apical spine and sometimes one to a few preapical spines. Plantula tiny; arolium large; claws asymmetrical and unspecialized. Cerci slender, tapered. Subgenital plate long, margin entire. Ovipositor long, nearly as half the length of the body, sabre-shaped, curved upwards distad.

### *Ensiferoblatta oecanthoides* sp. nov.

(Figs. 1–2, 3A–3C)

**Figure 1:**
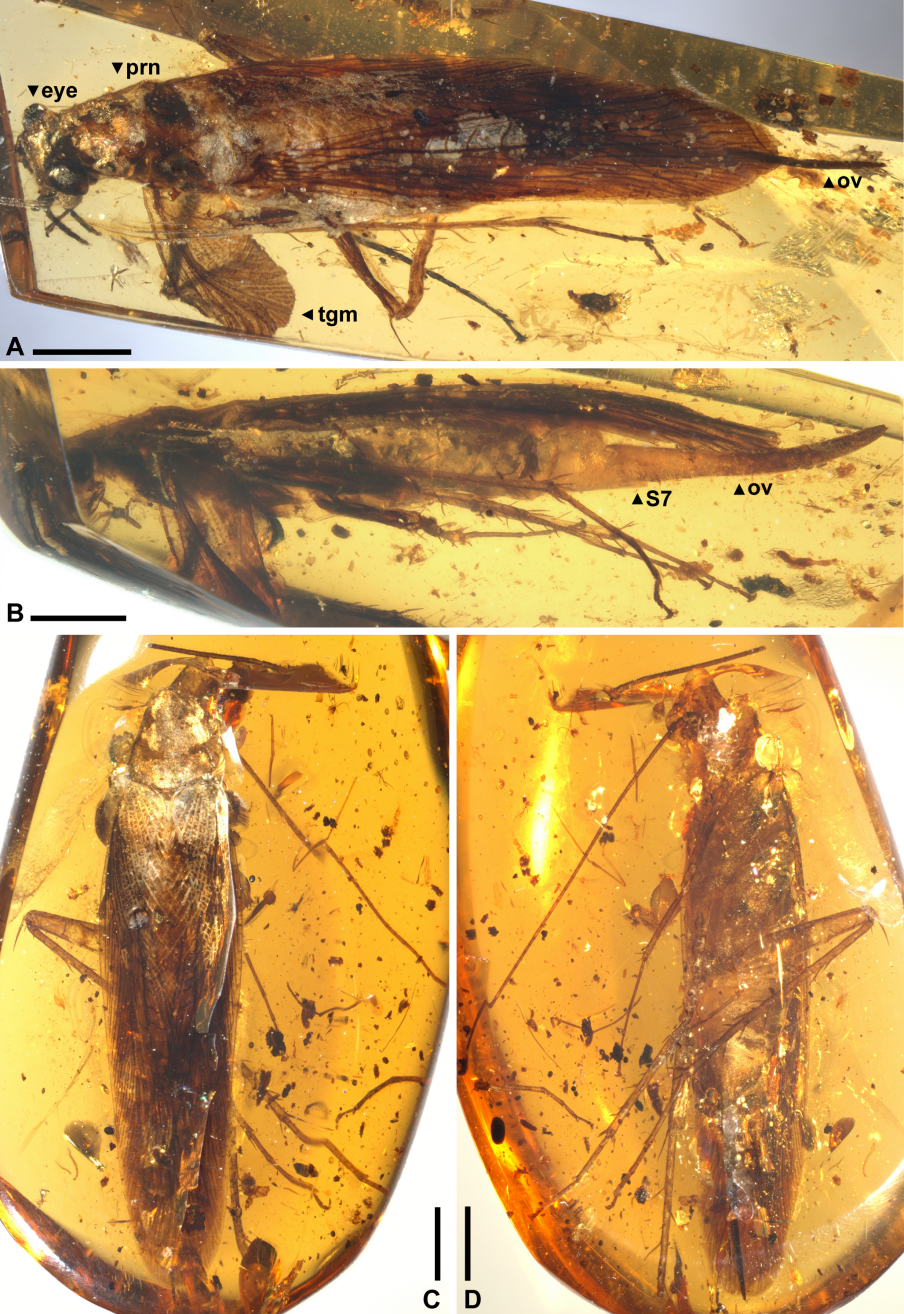
*Ensiferoblatta oecanthoides* gen. et sp. nov., habitus. (A–B) Holotype, NIGP200821, dorso-left view (A) and lateral view (B). (C–D) Paratype, NIGP200822, dorsal view (C) and ventro-left view (D). Abbreviations: ov, ovipositor; prn, pronotum; S7, seventh sternite; tgm, tegmen. Scale bars: 2 mm. Photo credit: Alan Hadley.

**Figure 2:**
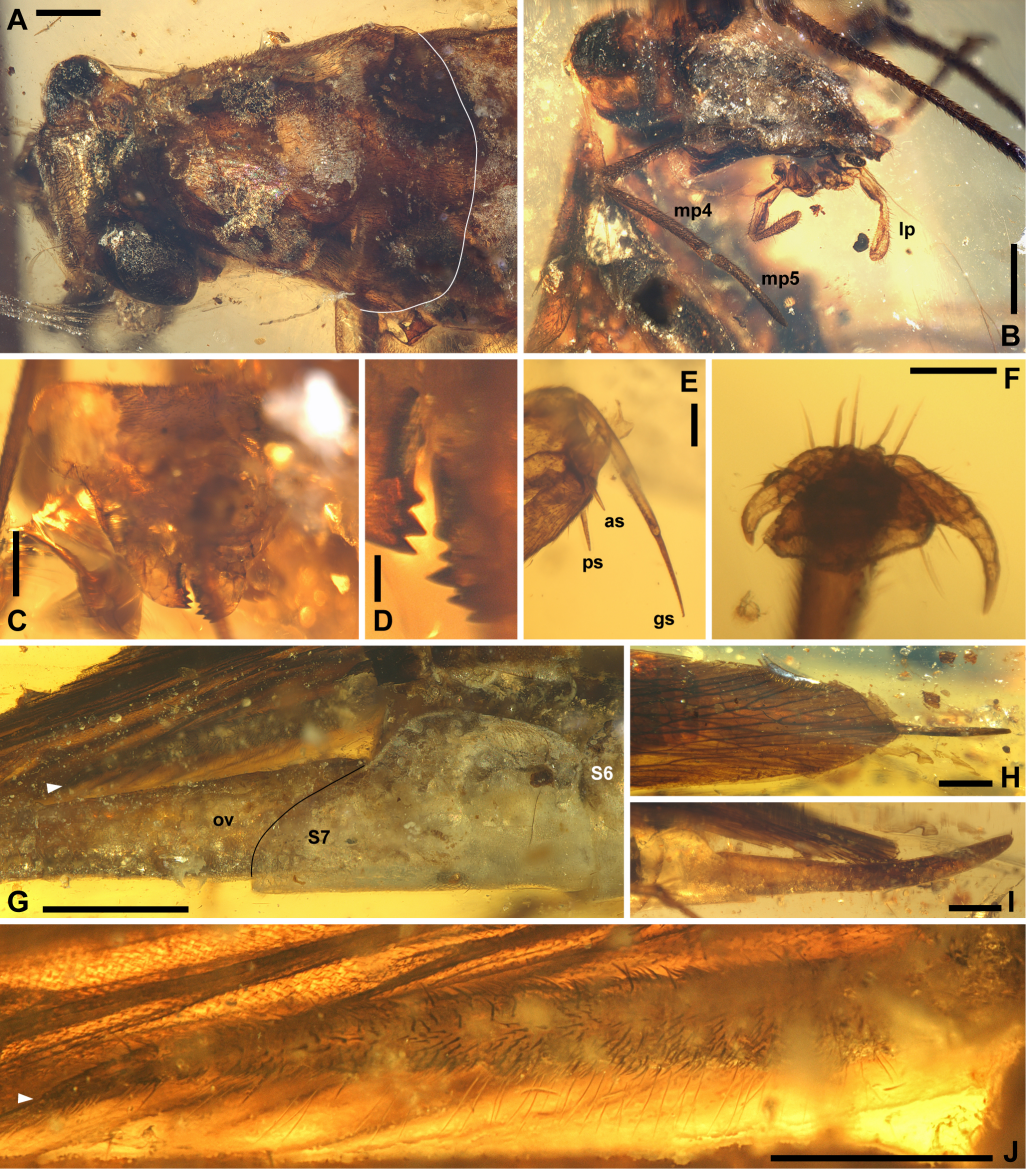
*Ensiferoblatta oecanthoides* gen. et sp. nov., details. (A) Head and pronotum of the holotype, NIGP200821, left-dorsal view, with a white line emphasizing the hind margin of pronotum. (B) Head of the holotype, view from mouthparts laterally. (C) Head of the paratype, NIGP200822, note that the light-coloured area is a damaged part, outlining the compound eye instead of being the eye. (D) Enlargement of the mandibles in C. (E) Articulation between femur and tibia of the hindleg of the paratype, anterior view (*i.e.,* ventral view of the specimen). (F) Pretarsus of the midleg of the holotype. (G) Terminalia of the holotype, with a black line emphasizing the margin of subgenital plate and a white arrowhead indicating the tip of cercus. (H–I) Dorsal and lateral views of the ovipositor of the holotype, to the same scale. (J) Cercus of the holotype, at the same view angle as G, with a white arrowhead indicating the tip of cercus. Abbreviations: as, anterior apical spine; gs, genicular spine; lp, labial palpus; mp4, mp5, maxillary palpomere IV and V; ov, ovipositor; ps, posterior apical spine; S6 and S7, sternum VI and VII. Scale bars: A, B, C, J, 500 µm; D, F, 100 µm; E, 200 µm; G, H, I, 1 mm. Photo credit: Alan Hadley.

**Figure 3:**
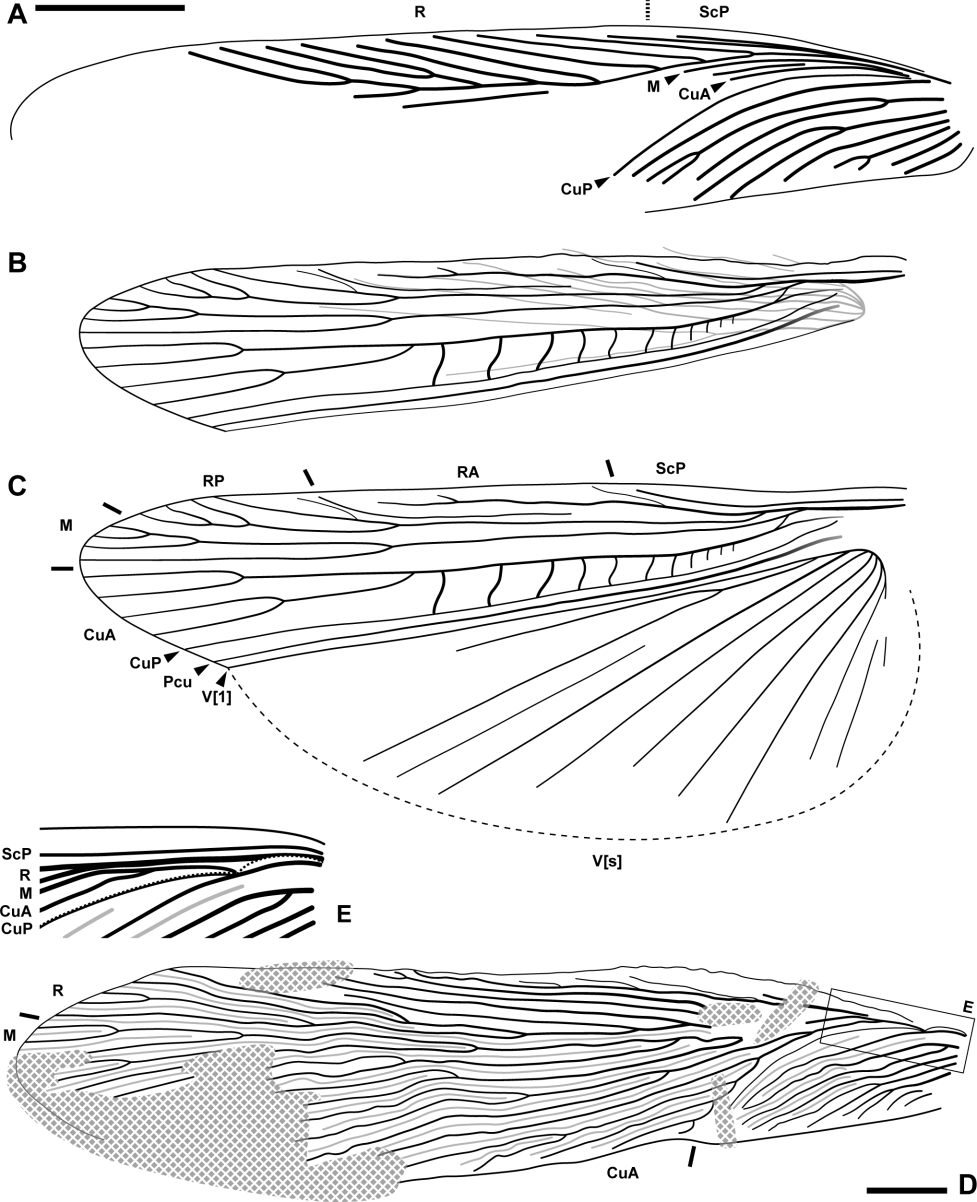
Wings of the fossils described herein. (A–C) *Ensiferoblatta oecanthoides* gen. et sp. nov., tegmen of the paratype, NIGP200822 (A) folded hindwing of the holotype, NIGP200821 (B) and reconstruction of B as unfolded (C) to the same scale. (D–E) Tegmen of *Proceroblatta colossea* gen. et sp. nov., holotype NIGP200823; the distorted basal portion is reconstructed and magnified by two times in E. Scale bars: 2 mm.

(LSID: urn:lsid:zoobank.org:act:2045B599-278B-457E-B580-93E3BBD39876)

**Material.** Holotype NIGP200821, female (Figs. 1A–1B), slightly decayed, nearly complete but most of the antennae, most of the left tegmen, and the distal part of left foreleg are missing. Paratype NIGP200822, female (Figs. 1C–1D), mostly preserved well, but distal part of antennae, most of right foreleg and right midleg, and distal part of the right hindleg are missing. In addition, a male nymph (NIGP200824) possibly belonging to this species is discovered, and this nymph bears some resemblance to *Raphidiomimula burmitica* Grimaldi and Ross, 2004 (Supplemental Information).

**Etymology.** Derived from *Oecanthus*, a genus of tree crickets; the general shape of the new species is reminiscent of *Oecanthus* (see Collins, Van den Berghe & Carson, 2014; Liu et al., 2018; Zefa et al., 2022).

**Diagnosis.** Only one species known; differential diagnosis unavailable.

**Description (female only).** Measurements of the paratype are given in parentheses, otherwise within the range of the holotype. General shape fusiform; body length ca. (11.8)–12.3, length including wings ca. 15.3–(16.8), length including ovipositor ca. (16.9)–17.3; shoulder width (between bases of forewings) ca. 2.2. Head: finely pubescent, width including eyes 2.03, length from vertex to apex of mandible (1.87); eyes rounded and bulbous, height 0.67, width 0.66, ocular distance at vertex 0.70; antennae (incompletely preserved) at least longer than half the body length; mandible normal as extant cockroaches (Fig. 2D); maxillary palpomere III/IV/V lengths 0.82/0.94/0.72; other characters of head indiscernible. Pronotum: finely pubescent, shape like a reversed shield, length ca. 2.62–(2.76) and width ca. 2.26–(2.37), ratio ca. 1.16; sides subducted. Wings (Figs. 3A–3C): forewing length ca. 12.9–(13.0) and width (2.53), ratio ca. (5.1), clavus length ca. (5.4) (longitudinal projection ca. 5.3) and width (1.51); ScP indiscernible; R with ten or 11 terminal branches recognized, proximal portion pectinate, middle portion with dichotomy; mediocubital veins largely indiscernible; CuP arcuate; claval veins essentially parallel to CuP, some of them bifurcate, terminating in 11 veinlets. Hindwing length 11.2, width of prevannus 2.22, length of vannal fold ca. 8.7; ScP/RA/RP/M/CuA with 1/3(or 4)/5(or 6)/4/4 terminal branches; CuA additionally with 11 incomplete branches that end at or approach CuP; CuP, Pcu and V[1] simple; base of V emitting eight V[s] veins and likely terminating in more than 11 veinlets. Leg segments length (femur/tibia//tarsomere I/II/III/IV/V): foreleg 2.70/2.68//1.38–(1.42)/(0.68)–0.76/0.44/0.18/0.36, midleg 3.70–(3.90)/3.28–4.24//1.56/0.66/0.40/0.14/0.40, hindleg 3.92–4.44/6.55–6.89//2.02–2.16/0.91–0.96/0.45–0.52/0.15/0.41. Each femur with a genicular spine, which is especially long in mid- and hindfemora. Forefemur with one apical spine and one preapical spine on both the anteroventral margin and the posteroventral margin. Midfemur similar to forefemur, but with four posteroventral spines, which are far apart. Hindfemur with two short apical spines respectively on the anteroventral and posteroventral margins, without preapical spines (Fig. 2E). Tibia with five distal long spines; additionally, foretibia with one anterodorsal, two anterovenral, one posterodorsal, and two posterovenral spines; midtibia with two anterodorsal, three anterovenral, two posterodorsal, and two posterovenral spines (nine spines arranged in four rows); hindtibia with three anterodorsal, four anterovenral, three posterodorsal, and two posterovenral spines (12 spines arranged in four rows). Plantula tiny, appearing to be paired lobes. Terminalia (Figs. 2G–2J): supra-anal plate damaged; cercus completely covered by wings, length 2.13, with 14 segments countable, segments longer distad; subgenital plate (sternum VII) convex, distal half roof-like, hind margin entire; ovipositor exposed length (5.22)–5.80 laterally and 5.12 ventrally, height at base 0.77, thickness (width) ca. 0.17, exceeding wings by (0.1)–2.08.

### Family undetermined

#### Genus *Proceroblatta* gen. nov.

(LSID: urn:lsid:zoobank.org:act:31D26EFE-086D-4228-A5F2-489BFADF3138)

**Type species.** *Proceroblatta colossea* gen. et sp. nov.

**Etymology.** Derived from Latin *procera*, extending to a great length, and *blatta*, cockroach. Feminine.

**Diagnosis (female only).** *Proceroblatta* at first sight closely resembles *Ensiferoblatta* with much larger size (ca. 2 times). It differs from *Ensiferoblatta* at least in very long maxillary palpi (length of palpomeres IV and V together is ca. 1.5X of head width, *vs.* ca. 0.8X in *Ensiferoblatta*), more spiny tibiae, and the longer cerci (exceeding *vs.* not exceeding wings). There might be more differences but the condition of preservation does not allow a further comparison.

### *Proceroblatta colossea* sp. nov.

(Figs. 3D–3E, 4)

**Figure 4:**
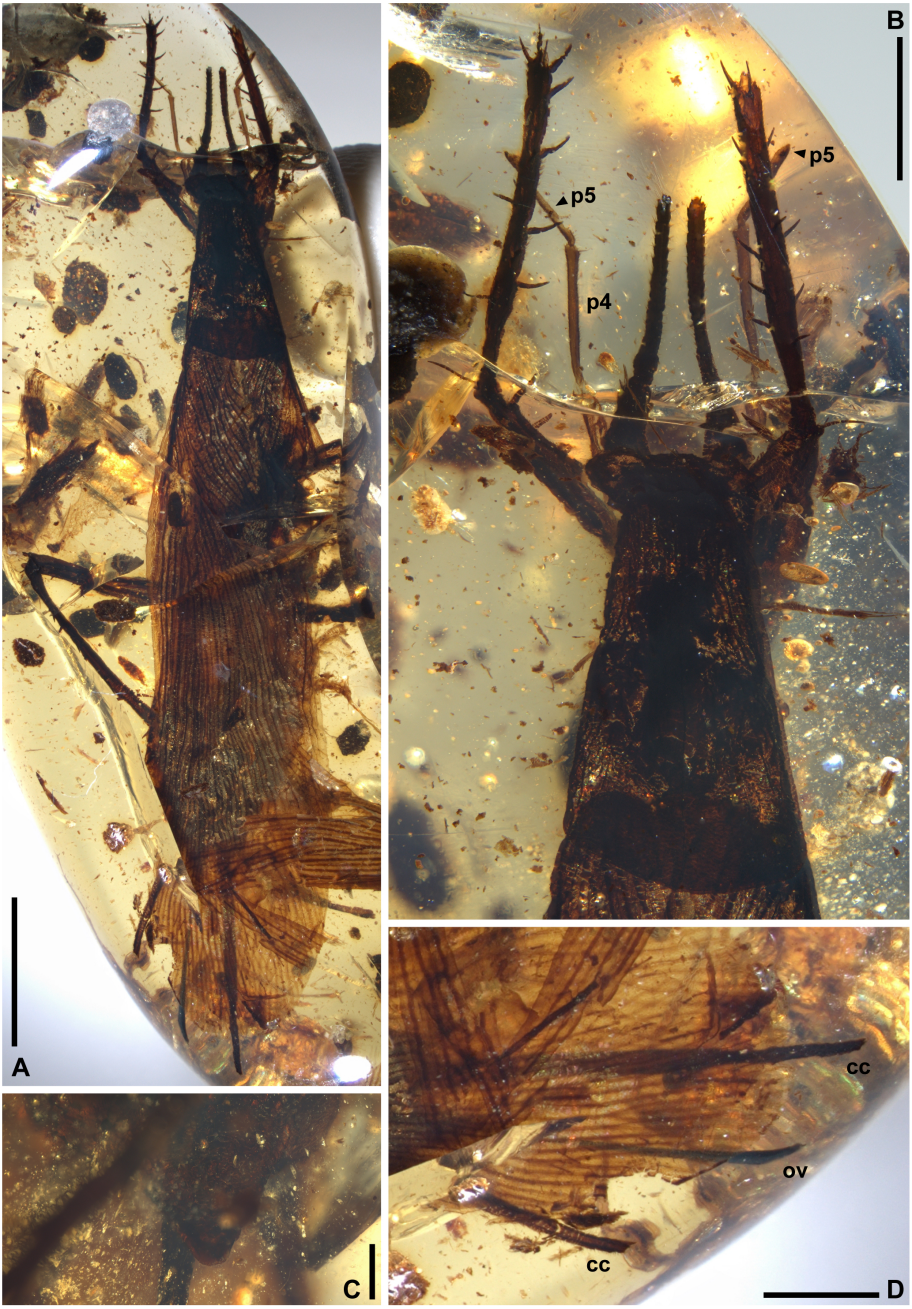
*Proceroblatta colossea* gen. et sp. nov., holotype NIGP200823. (A) Dorsal view of the habitus. (B) Details of the front portion, dorsal view. (C) Ventral view of the tip of subgenital plate. (D) Dorsal view of the terminalia, note that the cerci and ovipositor are truncated along with the amber piece. Abbreviations: cc, cercus; p4, p5, maxillary palpomere IV and V; ov, ovipositor. Scale bars: A, 5 mm; B,D, 2 mm; C, 500 µm. Photo credit: Alan Hadley.

(LSID: urn:lsid:zoobank.org:act:94C7A7F4-5A81-4498-904B-1D67C57BAE7B)

**Material.** Holotype NIGP200823, female (Fig. 4). The single fossil is preserved in a piece of Myanmar amber, decayed and distorted. Main parts are preserved but the following are missing: most of antennae, distal part of forelegs, most of midlegs, most of right hindleg, tarsus and pretarsus of left hindleg, and the distal part of cerci and ovipositor.

**Etymology.** Derived from Latin *colossea*, larger than usual.

**Diagnosis.** Only one species known; differential diagnosis unavailable.

**Description (female only).** General shape fusiform; body length 24.2, length including wings 29.3. Head not observed clearly, width ca. 2.4; eyes bulbous and seemingly rounded; maxillary palpi very long, the fourth palpomere length 1.96–2.12, the fifth segment length 1.32–1.63. Pronotum: long trapezoid, extremely fine pubescence seen along the margin (the surface hard to observe), hind margin arcuate, length 5.41 and width 3.42, ratio ca. 1.58. Wings (Figs. 3D–3E): forewing length ca. 23.9 and width 5.46, ratio ca. 4.38, clavus length ca. 7.1 (longitudinal projection ca. 6.6) and width 2.37; ScP seemingly simple; R essentially pectinate, with at least 11 terminal branches; mediocubital veins evenly distributed but unevenly dichotomous, with at least 19 terminal branches; CuP arcuate; claval veins essentially parallel to CuP, some of them bifurcate, terminating in ten branches; intercalary veins developed throughout. Hindwings indiscernible. Forefemur with at least three apical and preapical spines, but the insertion position unclear. Foretibia length at least 4.8 as preserved, distal spines incompletely preserved, additionally with two anterodorsal, four anterovenral, two posterodorsal, and three posterovenral spines. Hindfemur length greater than 5.8; hindtibia length greater than 14, with five distal spines, additionally with 22 spines, the arrangement of which is unclear. Terminalia: cercus thick, long, exceeding wings, length at least 7.5 as preserved (distal portion not included in the amber piece); subgenital plate (sternum VII) with triangular distal portion, hind margin entire. Ovipositor sword-shaped, exceeding wings (the apex not preserved), length at least 6.1 as preserved.

**Taxonomic placement of the new fossils.** With such a long external ovipositor, the new cockroach fossils are clearly not the members of Dictyoptera, to which extant cockroaches, termites and mantises belong. Cockroaches outside of Dictyoptera are placed into the paraphyletic plesiomorphon Eoblattodea (Laurentiaux, 1959) (see Li, 2019). The general shape of the new fossils is reminiscent of Raphidiomimidae, they all are slim, elongate cockroaches. However, Raphidiomimidae are prognathous and have fairly short, though exposed, ovipositors (see Vishnyakova, 1973), and these distinctions in key characters rule out a close relationship between the new fossils and Raphidiomimidae. Here we adopt the original concept of Raphidiomimidae by Vishnyakova (1973). In other words, questionable taxa in Raphidiomimidae are not taken into comparisons, *e.g.*, long-ovipositored species that are to be revised, and nymphs with limited discernible characters (Supplemental Information). Other long-ovipositored Mesozoic cockroaches were placed in Mesoblattinidae (Vishnyakova, 1968), with various length of ovipositor. Mesoblattinidae is a problematic taxon: it was based on *Blattina (Mesoblattina) protypa* Geinitz, 1880 (Handlirsch, 1906), the type specimen of which is merely a tegmen (Geinitz, 1880; Vršanský & Ansorge, 2007), and no topotype supplements the knowledge except for the tegmen. *Blattina (M.) protypa* has many traits in common with the general tegmen of extant Ectobiidae, *e.g.*, short ScP, pectinate R, somewhat irregular mediocubital veins, and diagonally distributed claval veins. The concept of “Mesoblattinidae” used by subsequent authors (*e.g.*, Vršanský & Ansorge, 2007) was abstracted from alleged confamilial species that were not ascertained to be closely related to *B. (M.) protypa*. In view of the current status of usage, the name Mesoblattinidae is confusing and thus undesirable. In summary, the new fossils cannot be placed in any known family.

We propose a new family for *Ensiferoblatta*, but the familial placement of *Proceroblatta* remains unspecified. The holotype of *Proceroblatta colossea* does not preserve adequate information for family-level classification. Fanwise folding of the hindwing was not detected in *Proceroblatta*. If *Proceroblatta* does not have fanwise folding, the hindwing would be an important difference with *Ensiferoblatta*. Noteworthily, the combination of long cerci and sword-shaped ovipositor is also found from some Mesozoic cockroaches, namely, *Karatovoblatta* and *Falcatusiblatta* (Vishnyakova, 1968; Liang, Shih & Ren, 2017). However, other characters of these genera do not exhibit relationships to *Proceroblatta*.

## Discussion

### Habitus, habit and habitat of the tree-cricket-like cockroaches

The typical body build of cockroaches is broad, flat, with transverse pronotum, and known Eoblattodea are no exception. In comparison, the new Eoblattodea cockroaches are slim, elongate, fusiform, with longitudinal pronotum. Apparently, *Ensiferoblatta* and *Proceroblatta* may be more adaptive to wide open habitats than to confined spaces (*e.g.*, plant litter, crevices and burrows), which are, as shelters, preferred by most extant cockroaches. Their habitus is reminiscent of fossil cockroach families Raphidiomimidae and Manipulatoridae, and extant cockroach genus *Saltoblattella*: Raphidiomimidae have elongate and prognathous head and might be predators (Vishnyakova, 1973; Vršanský, Vishniakova & Rasnitsyn, 2002; Grimaldi & Engel, 2005; Liang, Vršanský & Ren, 2012; Liang, Shih & Ren, 2017), Manipulatoridae have exceptionally long appendages (antennae and maxillary palpi in particular) and might be flower-visiting herbivores (Li & Huang, 2022; but see Vršanský & Bechly, 2015), and *Saltoblattella* is a jumping cockroach with well-developed saltatorial legs (Bohn et al., 2010). Their assumed habits have associations with open habitats. But on the other hand, the ethological and ecological hypotheses of them were not based on the body build alone, but on a comprehensive and comparative morphological investigation. *Ensiferoblatta* and *Proceroblatta* have long external ovipositors (thus belonging to Eoblattodea), the combination of such ovipositors and a fusiform instead of flat body build represents a unique morphotype among cockroaches. This morphotype resembles crickets and katydids (Ensifera, Orthoptera), especially the tree-crickets, more than general cockroaches.

Judging from the similarity with Ensifera in the ovipositor, Eoblattodea are not able to fabricate an ootheca of the dictyopteran type; instead, they likely lay eggs one by one into ground or plant material, as Ensifera. The shape of ovipositor is indictive of the substrate for Ensifera to lay eggs: straight, cylindrical or needle-like ones suggest soil, while curved, sickle-like ones suggest plant material (Rentz, 2010). The ovipositor of *E. oecanthoides* is sabre-like, and its tip is not serrated. The preserved ovipositor portion of *P. colossea* is similar to that of *E. oecanthoides*. This type of ovipositor may indicate the hollow grass stems as oviposition site (Rentz, 2010). In extant Ensifera, the meadow katydids (*Conocephalus*) have suchlike ovipositors (see Nagar & Swaminathan, 2016; Farooqi & Usmani, 2018). They lay eggs in the leaf sheaths of a variety of grasses, reeds and sugar cane, occasionally in stems, wood cracks, and even in fruits (Illingworth, 1931; Zimmerman, 1948; Powell, 1977; Decleer, 1991; Divya & Senthilkumar, 2018). Accordingly, *E. oecanthoides* and *P. colossea* likely lay eggs in similar substrate. Besides, their ovipositors *per se* are not adaptive for fossorial purpose, unlikely penetrating sand, soil or hard stems of plants, but suitable for flimsy substrate. However, even if a few grasses had coexisted with *E. oecanthoides* and *P. colossea*, grasses had not been common until the end of Cretaceous at least (Soreng et al., 2015; Kirschner & Hoorn, 2020). Therefore, the oviposition site should be of other plants with suitable properties. Note that the external ovipositors of Eoblattodea, including *E. oecanthoides* and *P. colossea*, are distinct from that of extant mantises and extinct basal Dictyoptera. The slightly exposed ovipositors of the latter are too short to lay eggs as deeply as effective for protection, the valvulae of some species are too soft and too loose to cut or penetrate (see Bai et al., 2018; Qiu, Wang & Che, 2019); instead, their ovipositors correlate to certain oothecae typical of mantises (see Li & Huang, 2019).

The asymmetrical tarsal claws may have latent implication on the habit of *Ensiferoblatta*. Asymmetrical claws are found among extant cockroaches (*e.g.*, Roth, 1993; Deans & Roth, 2003; Bohn & Chládek, 2011; Anisyutkin, 2012; Li et al., 2017; Qiu et al., 2017), but the natural history of only few taxa has been observed. Many Polyzosteriinae have asymmetrical claws (Roth, 2003), and these cockroaches frequently inhabit heath and deserts and associated with xeric plants in Australia (Rentz, 2014). Some species of the European *Phyllodromica* Fieber, 1853 (*maculata*-group) are thermophilic and often found in the grassy vegetation on southerly exposed slopes of hills (Bohn & Chládek, 2011). Species of *Sorineuchora* Caudell, 1927 from the monsoon forests in South China are frequently found on coarse bark of trees, while sympatric cockroaches mainly occur in shrubs, leaf litter and crevices (XRL pers. obs.). Females of *Nyctibora acaciana* Roth, 2003 from Costa Rican dry forest were observed gluing their oothecae to the stems and branches of ant-acacias (Deans & Roth, 2003). Apparently, cockroaches with asymmetrical tarsal claws favour a specific texture (especially that of a certain plant), or the claws resulted from the adaptation of such texture. However, the underlying correlation and the functional mechanism are unclear.

**Figure 5:**
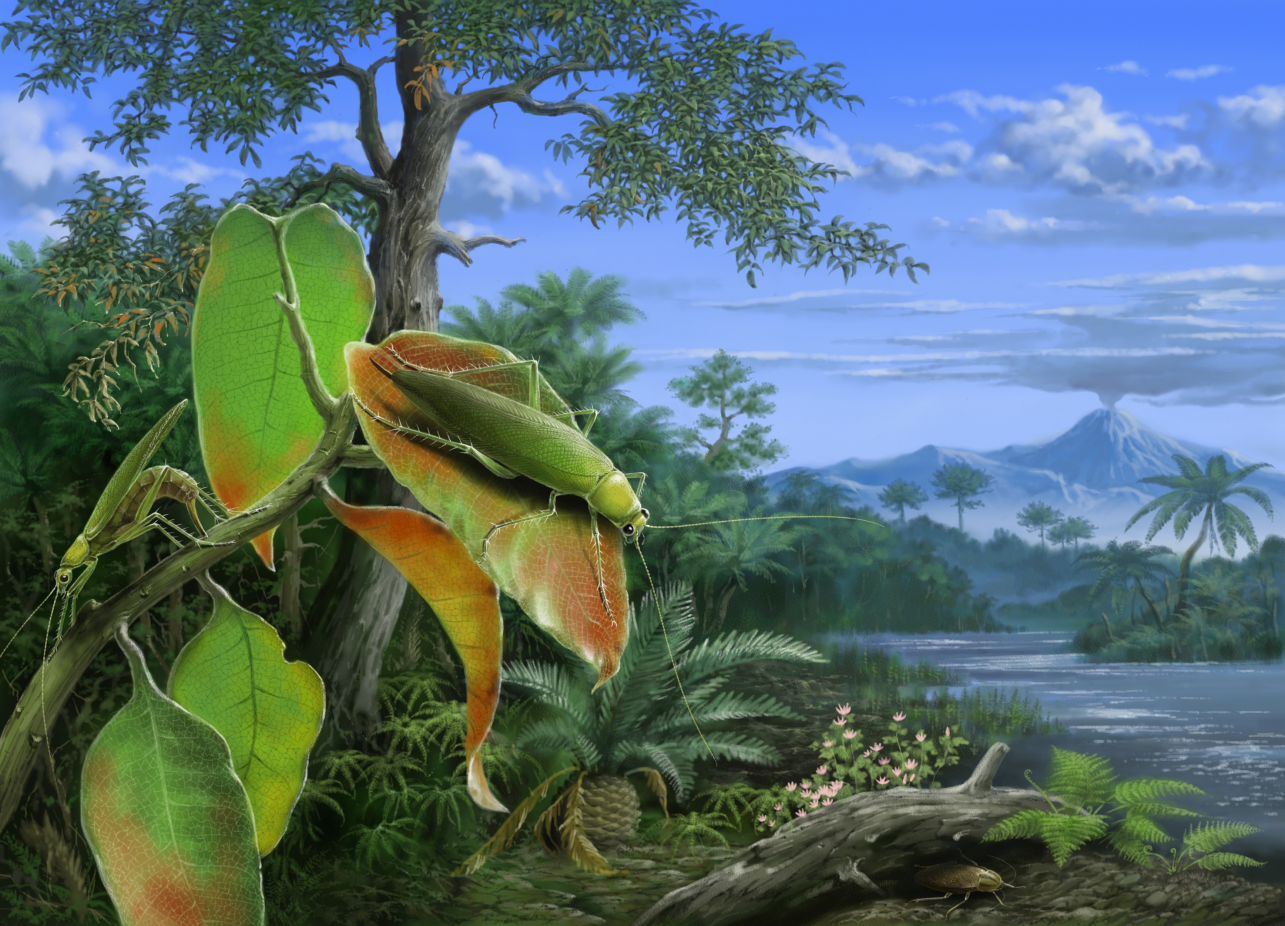
Ecological reconstruction of *Ensiferoblatta oecanthoides* gen. et sp. nov. This picture shows two females on a twig of an angiosperm, and one of them is laying eggs into the stem. Image credit: Mr Jie Sun.

Current fossil records suggest that *Ensiferoblatta* and *Proceroblatta* represent a new morphotype that did not exist until the middle-late Mesozoic. Provided that it is a fact, some Eoblattodea might have entered a new niche during Jurassic or Cretaceous, and this niche may be associated with a contemporary transformation of ecosystems. During the middle-late Mesozoic, the rapid radiation of angiosperms reshaped the terrestrial ecosystems, and led to the co-diversification of insects by opening new niches (Labandeira, 2014; Li et al., 2019; Benton, Wilf & Sauquet, 2021). The morphotype represented by the new fossils seems like an evolutionary attempt of the ancient Eoblattodea, interacting with the newly emerged angiosperms. In summary, *Ensiferoblatta* and *Proceroblatta* may be arboreal insects, probably feeding on certain angiosperms, and these unique cockroaches may also lay eggs into penetrable stems of those plants, or prepare the stem by biting before oviposition (Fig. 5).

### Fossil records and extinction of cockroaches with long ovipositor

A cockroach with long external ovipositor (Eoblattodea, also known as ‘roachoid’) is not found in recent fauna. It is believed that this type of cockroaches became extinct by Cenozoic, but little is known about how far they went. Long, stiff, external ovipositors were occasionally documented in fossils from latest Carboniferous (sword-shaped or simply spinous: Laurentiaux, 1951; Laurentiaux, 1959; Bekker-Migdisova, 1962) to Early Cretaceous (sword-shaped: Vishnyakova, 1986; Ren et al., 1995, therein dated to Jurassic), remarkably in the Jurassic (Vishnyakova, 1968; Liang, Shih & Ren, 2017). New fossils described herein demonstrate that the history of this type of cockroaches is longer than formerly known. The long history of such ovipositor suggests that this morphotype used to be very successful for cockroaches.

The most conspicuous, and likely the most significant, difference between earlier and extant cockroaches lies in the ovipositor, or in other words, in the absence and presence of the oothecae. It leads to a straightforward conclusion that the absence of oothecae contributes to the extinction of Eoblattodea, because the eggs lack proper protection so as to be vulnerable. Interestingly, crickets, katydids and ootheca-producing cockroaches finally survive, whereas the cockroaches bearing long ovipositors typical of crickets and katydids died out. Besides, the eggs of Eoblattodea could be protected as well as that of Ensifera, providing that these insects lay eggs into similar substrates. Therefore, the ‘old-fashioned’ style of oviposition may not be the key contributor to the extinction of Eoblattodea.

Here we explore the cause of their extinction in the context of fossil records and ecological changes. As mentioned above, the ovipositors of cockroaches evolved towards reduction during middle-late Mesozoic, as shown by fossil records. This marks the decline of Eoblattodea and the origin of Dictyoptera and Blattodea. Together with the new morphotype of Eoblattodea, at least three evolutionary paths of the last Eoblattodea are revealed: (1) to retain the traditional morphotype and habits, (2) to retain the long ovipositor and ancient oviposition strategy, but develop new morphotypes to adapt to new niches, and (3) to shorten the ovipositor so as to fabricate an ootheca, giving rise to Dictyoptera (please note that Eoblattodea is a character-based paraphyletic taxon, and Dictyoptera is one of its descendants but is not taxonomically subordinate to Eoblattodea. See Li, 2019). The first and second paths resulted in dead ends, while the third is an open path. In view of the timing, an apparent relation is readily spotted between the extinction of Eoblattodea and the end-Cretaceous mass extinction event. However, in spite of a decrease in the estimated diversity of insects during the Late Cretaceous (Clapham et al., 2016; Schachat et al., 2019), insects seemed to have not suffered a severe decline from the end-Cretaceous event, and the insect diversity of this epoch is likely underestimated owing to the lack of Lagerstätten (Schachat & Labandeira, 2021). Instead, the dynamics of insects are more closely associated with the Angiosperm Terrestrial Revolution: while angiosperms gradually replaced gymnosperms as the dominant plants, a number of plant-associated insects shifted their hosts from gymnosperms to angiosperms, others remained in gymnosperms or became extinct (Labandeira, 2014). Accordingly, we conjecture the evolutionary scenario of middle-late Mesozoic Eoblattodea: they might utilize a certain group of gymnosperms for feeding or egg-laying, and the majority of them failed to adapt to new host plants before the existing hosts became extinct (path 1), a few species managed to live on new, angiosperm hosts, but also failed in the end (path 2), and a small group of Eoblattodea no longer retained a long ovipositor, they did not rely on gymnosperms anymore and probably entered a niche distinct from that of other Eoblattodea (path 3). From the perspective of geological history, the candidates for the hosts of the last ‘conventional’ Eoblattodea include Bennettitales: these cockroaches and gymnosperms both originated in the late Palaeozoic and severely declined during the Late Cretaceous (Stewart, 1983; Knoll, 1986; Blomenkemper et al., 2021). Similarly, certain subgroups of Ginkgoales and Cycadales are also possible. The extinction of the new morphotype of Eoblattodea might be due to the small population and a low degree of adaptive flexibility. In terms of the assumed habit—being exposed to open environment, *Ensiferoblatta* and *Proceroblatta* were readily affected by ecological disturbance and vulnerable to catastrophic events. Moreover, like ‘conventional’ Eoblattodea, *Ensiferoblatta* and *Proceroblatta* also left their eggs to the habitat, and the eggs risked more being eaten or impaired. In contrast, cockroaches that tend to hide in crevices and burrows, like many extant species, have a higher chance of survival. In addition, the oothecae of extant cockroaches are a sound, maternal protection for the eggs. Running on a lucky evolutionary path, cockroaches that produce oothecae with shortly exposed or internal ovipositors (together included in Dictyoptera) took this opportunity to diversify, and became the cockroaches, mantises and termites today, which distribute throughout tropical and subtropical regions.

## Conclusions

This article reports the discovery of *Ensiferoblatta oecanthoides* gen. et sp. nov. and *Proceroblatta colossea* gen. et sp. nov. from mid-Cretaceous Myanmar amber. These new taxa belong to the ancient group of cockroaches, namely Eoblattodea (also known as ‘roachoids’), which date back to the Carboniferous. They are the youngest Eoblattodea ever found, extending the history of Eoblattodea as long as over 200 My, demonstrating the evolutionary triumph of the cockroaches with long ovipositor, which lay eggs into plants and soil instead of fabricating an ootheca. The tree-cricket-like morphotype of *Ensiferoblatta* and *Proceroblatta* is unique among Eoblattodea and also rare among cockroaches, and indicates that these cockroaches preferred wide open habitats, instead of confined spaces that ‘traditional’ cockroaches shelter in. This unique morphotype appears like an attempt to adapt to newly emerged angiosperms, but resulted as an evolutionary dead end. The morphotype and habit might contribute to the extinction of *Ensiferoblatta* and *Proceroblatta*, because the open habit causes latent impairment to viability. We also speculate that the extinction of ‘traditional’ Eoblattodea is probably due to the extinction of their gymnosperm hosts. The lack of protection for eggs (maternal care in particular) might accelerate the extinction of Eoblattodea as a whole.

##  Supplemental Information

10.7717/peerj.15067/supp-1 Supplemental Information 1 Supplemental Information

## Additional Information and Declarations

### Data Availability

The following information was supplied regarding data availability: This article focuses on the description of new fossils, accessioned as specimen numbers NIGP200821–200824, at Nanjing Institute of Geology and Palaeontology, Chinese Academy of Sciences, Nanjing, Jiangsu, China.

### New Species Registration

The following information was supplied regarding the registration of a newly described species: Ensiferoblattidae: urn:lsid:zoobank.org:act:A0CC40E4-8990-4CA5-9854-D1670E93EB11 Ensiferoblatta: urn:lsid:zoobank.org:act:EA84E47F-47AD-4F79-AFF6-929484FCDDF3 Ensiferoblatta oecanthoides: urn:lsid:zoobank.org:act:2045B599-278B-457E-B580-93E3BBD39876 Proceroblatta: urn:lsid:zoobank.org:act:31D26EFE-086D-4228-A5F2-489BFADF3138 Proceroblatta colossea: urn:lsid:zoobank.org:act:94C7A7F4-5A81-4498-904B-1D67C57BAE7B Publication LSID: urn:lsid:zoobank.org:pub:7015F012-0008-4885-BB51-C1340A0A3CA2
