# Supplementary material for: Atypical ‘long-tailed’ cockroaches arose during Cretaceous in response to angiosperm terrestrial revolution
Source: PeerJ. 2023 Mar 29;11:e15067. doi: 10.7717/peerj.15067 (PMC10066690; doi:10.7717/peerj.15067)
Supplement: Supplemental Information 1 [file peerj-11-15067-s001.pdf]

Supplemental Information for

Atypical 'long-tailed' cockroaches arose during Cretaceous in response to angiosperm terrestrial revolution

by

Xin-Ran Li and Di-Ying Huang

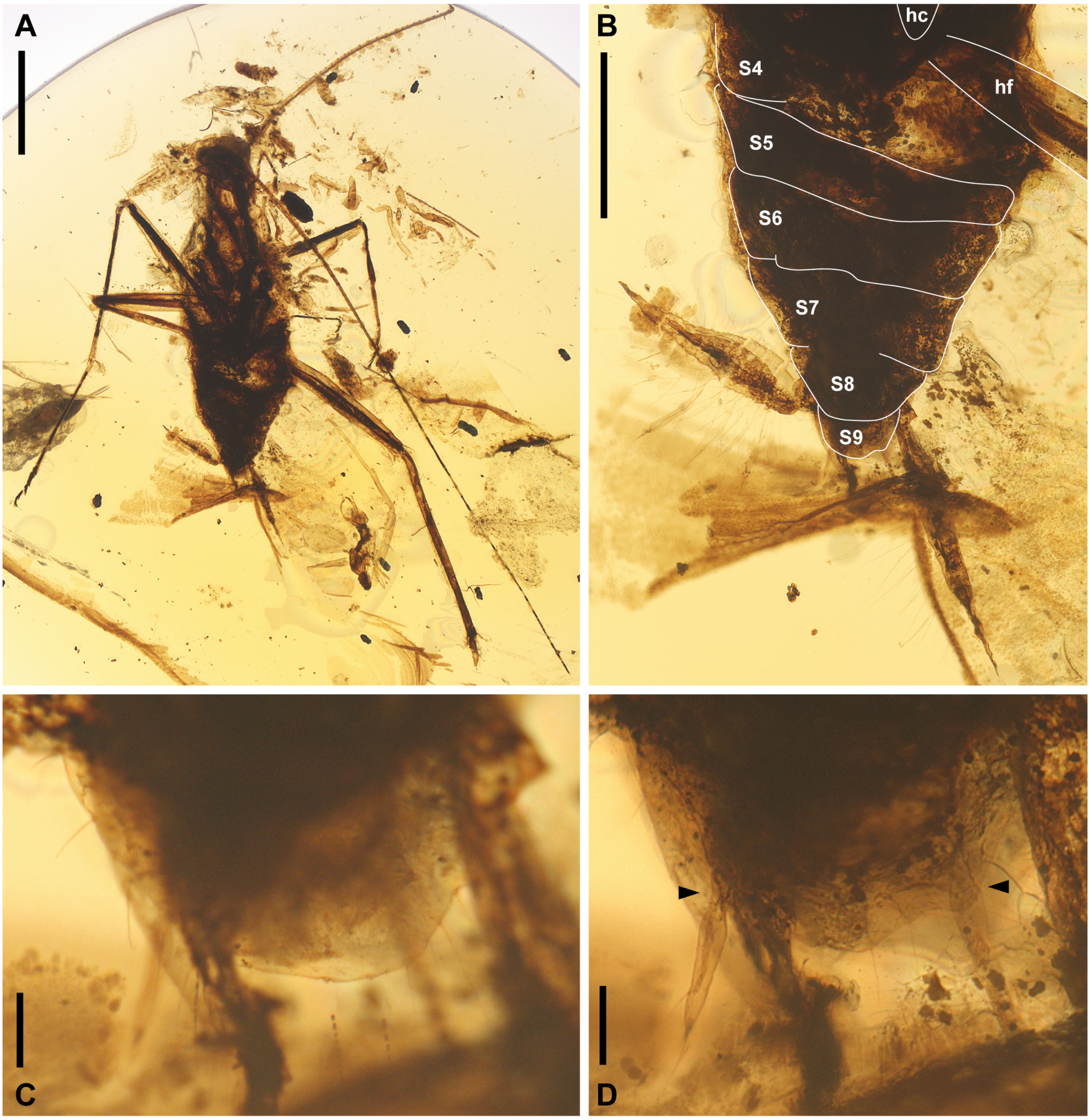

Figure S1. An undetermined male nymph, NIGP200824, possibly *Ensiferoblatta* sp. **A.** Ventral view. **B.** Ventral view of the abdomen, with white lines emphasizing the margins of sterna and of the leg. **C.** Tergum X (supra-anal plate) in ventral view. **D.** Sternum IX (subgenital plate) in ventral view, with black arrowhead indicating the insertions of styli. Abbreviations: hc, hindcoxa; hf, hindfemur; S4–S9, sternum IV through IX. Scale bars: A, 2 mm; B, 1 mm; C,D, 100 µm.

Text S1

Description of the male nymph, NIGP200824

Specimen badly decayed. Body fusiform, length 6.6 mm, with the abdomen accounting more than a half (ca. 3.5 mm). Head almost completely exposed, not covered by the elongate trapezoid pronotum; antennae much longer than body. Legs long, length of segments (femur/tibia//tarsomere 1/2/3/4/5): foreleg 1.9–2.2/1.2//0.9/0.3/0.1/0.1/0.2, midleg 2.5/2.2//?, hindleg 3.4–3.6/3.2–3.8//1.8/0.6/0.2/0.1/0.3. Hind margin of tergum X (supra-anal plate) arcuate. Cerci fusiform, acuminate, with long hairs, length ca. 1.4–1.7. Sternum IX (subgenital plate) small, nearly symmetric (appearing asymmetric in Fig. S1D due to preservation condition). Two similar styli far apart, not specialized. Possible hook-like phallomere on the right.

Remarks. This specimen accords with the general morphology of *Ensiferoblatta* and *Proceroblatta*, and more similar to the former in the smaller size, shorter maxillary palpi, and shorter cerci. It is possibly an *Ensiferoblatta* sp., or at least a closely related genus. This male nymph bears some resemblance to *Raphidiomimula burmitica* Grimaldi and Ross, 2004, at least in the fusiform body, slightly elongate maxillary palpi, elongate pronotum, and long, fusiform cerci. However, key characters such as ocelli and fine details of terminalia are indiscernible in both specimens, and a nymph bears too limited information for comparison and taxonomy. Therefore, the relationship between these specimens is not clear and both specimens are not determined with confidence to a specific higher taxon. *Raphidiomimula burmitica* was originally placed in Raphidiomimidae (Grimaldi and Ross, 2004), but subsequently excluded (Gorokhov, 2007; Liang et al., 2009). The opisthognathous head of *R. burmitica* is too distinct from the prognathous head of Raphidiomimidae, and the comparatively short foretibiae are also unusual for Raphidiomimidae. On the other hand, the resemblance between *R. burmitica* and NIGP200824 might suggest a close relationship between *Raphidiomimula* and *Ensiferoblatta*.

References

Gorokhov AV. 2007. New and little known orthopteroid insects (Polyneoptera) from fossil resins: communication 2. Paleontological Journal 41(2): 156–166.

Grimaldi DA, Ross AJ. 2004. *Raphidiomimula*, an enigmatic new cockroach in Cretaceous amber from Myanmar (Burma) (Insecta: Blattodea: Raphidiomimidae). Journal of Systematic Palaeontology 2(2): 101–104.

Liang J-H, Vršanský P, Ren D, Shih C. 2009. A new Jurassic carnivorous cockroach (Insecta, Blattaria, Raphidiomimidae) from the Inner Mongolia in China. Zootaxa 1974(1): 17–30.
